# Massive Blood Transfusion for Trauma Score to Predict Massive Blood Transfusion in Trauma

**DOI:** 10.1155/2021/3165390

**Published:** 2021-02-24

**Authors:** Osaree Akaraborworn, Boonying Siribumrungwong, Burapat Sangthong, Komet Thongkhao

**Affiliations:** ^1^Division of Trauma and Critical Care, Department of Surgery, Faculty of Medicine, Prince of Songkla University, Hat Yai, Thailand; ^2^Division of Vascular and Endovascular Surgery, Department of Surgery, Faculty of Medicine, Thammasat University Hospital, Khlong Nueng, Pathum Thani, Thailand

## Abstract

**Background:**

Massive blood loss is the most common cause of immediate death in trauma. A massive blood transfusion (MBT) score is a prediction tool to activate blood banks to prepare blood products. The previously published scoring systems were mostly developed from settings that had mature prehospital systems which may lead to a failure to validate in settings with immature prehospital systems. This research aimed to develop a massive blood transfusion for trauma (MBTT) score that is able to predict MBT in settings that have immature prehospital care.

**Methods:**

This study was a retrospective cohort that collected data from trauma patients who met the trauma team activation criteria. The predicting parameters included in the analysis were retrieved from the history, physical examination, and initial laboratory results. The significant parameters from a multivariable analysis were used to develop a clinical scoring system. The discrimination was evaluated by the area under a receiver operating characteristic (AuROC) curve. The calibration was demonstrated with Hosmer–Lemeshow goodness of fit, and an internal validation was done.

**Results:**

Among 867 patients, 102 (11.8%) patients received MBT. Four factors were associated with MBT: a score of 3 for age ≥60 years; 2.5 for base excess ≤–10 mEq/L; 2 for lactate >4 mmol/L; and 1 for heart rate ≥105 /min. The AuROC was 0.85 (95% CI: 0.78–0.91). At the cut point of ≥4, the positive likelihood ratio of the score was 6.72 (95% CI: 4.7–9.6, *p* < 0.001), the sensitivity was 63.6%, and the specificity was 90.5%. Internal validation with bootstrap replications had an AuROC of 0.83 (95% CI: 0.75–0.91).

**Conclusions:**

The MBTT score has good discrimination to predict MBT with simple and rapidly obtainable parameters.

## 1. Introduction

Trauma is one of the leading causes of death globally. In 2013, trauma accounted for 4.8 million deaths worldwide [1]. Hemorrhage was the most common cause of traumatic death in the first 24 hours [2]. It was found that timely resuscitation with blood components with a ratio of packed red blood cells (PRBCs) to fresh frozen plasma (FFP) between 1 : 1 and 2 : 1 decreased the mortality rate from exsanguination [3]. A massive blood transfusion (MBT) protocol is a preset guideline between the clinicians and the blood bank to prepare blood products in a timely manner. Therefore, MBT protocols effectively help to reduce unnecessary blood component usage [4].

Usually, the MBT protocol is triggered by MBT scores derived from clinical parameters, such as low blood pressure or tachycardia. Many MBT scores have been published and validated [4]; however, the scores had good prediction only when they were applied in the same settings where the scores were created [5]. For instance, the assessment of blood consumption (ABC) score, which is one of the popular scores, had good prediction performance with an AuROC of 0.842 in the original setting but had an AuROC of 0.587 when validated in Thailand, which was probably caused from different mechanisms of injury of patients [6, 7].

In addition, most MBT scores were created from well-developed countries with mature trauma systems and advanced prehospital care. However, some parameters, such as radiographic imaging, cannot be obtained on a timely basis in developing countries as in Thailand. The majority of prehospital care providers in Thailand cannot deliver advanced prehospital resuscitation which may lead to delayed resuscitation and deterioration of the hemodynamics by the time the patients arrive at the hospital. Therefore, the cut points of the parameters used in well-developed countries may not be suitable in developing countries due to the limitation of prehospital management. This study aimed to create an MBT score to predict MBT in a setting that does not have advanced prehospital care.

## 2. Materials and Methods

### 2.1. Design and Setting

This study was a diagnostic prediction study conducted in Songklanagarind Hospital which is a university-based hospital and a level 1 trauma center in Southern Thailand. Annually, Songklanagarind Hospital has about 10,000 trauma patients who visit the emergency department with about 1600 trauma admissions. Only 50% of the patients were transported to the emergency department by the basic life support team [8]. This study was approved by the ethics committee of the Faculty of Medicine, Prince of Songkla University.

### 2.2. Population

The enrolled subjects were adult trauma patients aged ≥15 years who met at least one of the criteria for activating the trauma team of Songklanagarind Hospital within 1 hour after admission from January 2012 to December 2018. The criteria of the trauma activation protocol are (1) systolic blood pressure (SBP) ≤90 mmHg; (2) gunshot wound (GSW) at the chest, abdomen, or back; (3) stab wound at the chest or abdomen or both; (4) respiratory rate <12/min or >30/min; (5) heart rate (HR) >120/min; (6) Glasgow Coma Scale (GCS) score ≤8; and (7) a positive Focused Assessment Sonography in Trauma (FAST) exam. Patients were excluded if they were dead on arrival, injured from hanging or burns, or had missing primary outcome data due to incomplete medical records. Patients who died from exsanguination but did not receive MBT were also excluded from the study.

### 2.3. Outcomes

MBT was defined as a patient who received PRBC >10 units within 24 hours or >4 units within 1 hour [9, 10].

### 2.4. Predictive Parameters

Only parameters that could be obtained within a few minutes after arrival were included in the study. Individual parameters such as age and gender were also collected. Physiological parameters consisted of vital signs and GCS score. Injury parameters consisted of the mechanism of injury (i.e., road traffic injury, GSW, stab wound, and fall), pelvic and femur fractures that were diagnosed by physical examination, and the presence of free fluid from the FAST examination. Laboratory results were blood lactate and base excess. Hematocrit results were not included in the study because in the local setting, hematocrit results take longer to obtain.

### 2.5. Data Collection Process

The data were collected and were retrieved from the trauma registry and the hospital electronic medical records of Songklanagarind Hospital.

### 2.6. Sample Size

According to the TRIPOD guideline [11] for a predictive model, at least 10 events are required for one candidate variable. Eight variables were estimated to be included in the final model. A previous study at our institute demonstrated that the incidence of MBT was 9% in patients activated by the activation protocol [7]. Therefore, the sample size was estimated to be about 889 patients for 80 events.

### 2.7. Statistical Methods

Continuous variables are presented as mean and standard deviation or median and range according to the distribution of the data. Categorical variables are expressed as frequency and percentage. Univariable analysis was performed, and the candidate predictors identified as variables with significant values (*p* < 0.20) in the univariable analysis, as well as clinically important variables, were selected for a multivariable logistic regression analysis [12]. Backward elimination was then conducted to identify a parsimonious model. The score was then derived from complete data only. Two-tailed *p* values <0.05 indicated a significant difference. The coefficients of the significant variables from multivariable analysis were used to generate risk scores. Sensitivity, specificity, and positive likelihood ratio (LR+) were also demonstrated. The cut point of the score was calibrated from LR+. Discrimination was demonstrated by AuROC. The Hosmer–Lemeshow calibration test was used to determine the concordance between prediction and observations. Internal validation was done with bootstrap replications.

## 3. Results

**Figure 1:**
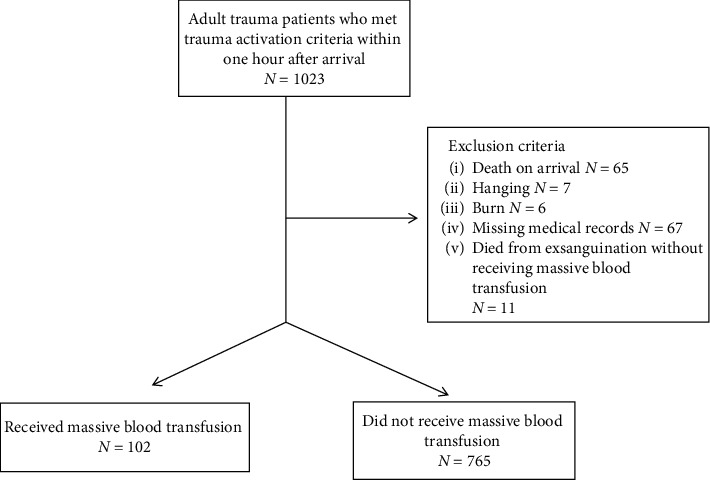
Diagram of patient enrollment.

During the seven years of the cohort period from 2012 to 2018, 1023 patients met the eligibility criteria. One hundred and fifty-six patients were excluded because of death on arrival, hanging, burn, and missing medical records, and 11 patients did not receive MBT but died from exsanguination. Therefore, the remaining 867 patients were included in the study. The patients who were entered into the study are shown in Figure 1.

Among the 867 patients, 102 patients (11.8%) were in the group that received MBT, and the remaining 765 (88.2%) patients were in the non-MBT group. Most of the patients in both groups sustained motorcycle crash injuries (54.9% vs. 44.6%). The group that received MBT had a higher median injury severity score (ISS) (32 vs. 14, *p* < 0.001). The median number of transfusion units of PRBC in the MBT group was 17 units (IQR: 13–25), while the non-MBT group had a median number of transfusion units of PRBC of 0 (IQR: 0–2). The results of the univariable analysis are shown in Table 1. From the univariable analysis, 11 predictors were included in the multivariable model. Those predictors were age ≥60 years, GCS score ≤8, SBP ≤90 mmHg, GSW, HR ≥ 105/min, presence of pelvic fracture from physical examination, presence of femur fracture from physical examination, need for nasal packing for hemostasis, BE ≤ –10 mEq/L, lactate >4 mmol/L, and presence of free fluid from FAST.

### 3.1. Score Derivation

Multivariable logistic regression showed four parameters that remained in the model: age ≥60 years old (coefficient: 2.33, *p* < 0.001), BE ≤ –10 mEq/L (coefficient: 1.70, *p* < 0.001), lactate >4 mmol/L (coefficient: 1.42, *p* = 0.001), and HR ≥ 105/min (coefficient: 0.74, *p* = 0.04). The prediction risk of MBT can be written as the following equation: prediction risk of MBT = –4.39 + 2.33*x* (age ≥60) + 1.7*x* (BE ≤ –10 mEq/L) + 1.42*x* (lactate > 4 mmol/L) + 0.74*x* (HR ≥ 105/min). The coefficients were used to calculate the prediction score. The weightings of age, BE, lactate, and HR were 3, 2.5, 2, and 1, respectively. The derivations of the scores are shown in Table 2.

### 3.2. Score Performance

**Figure 2:**
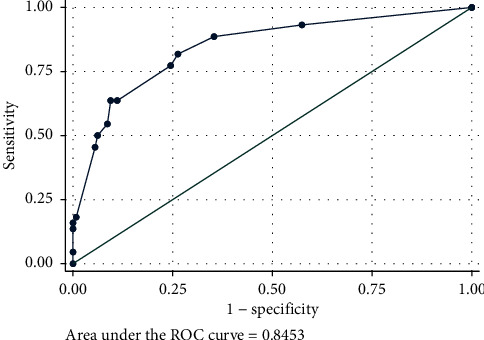
AuROC of the MBT score.

**Figure 3:**
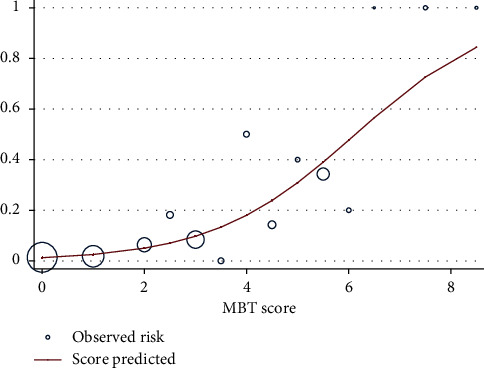
Observed and predicted rates for MBT for each MBT score. Size of the circle represents the population size for each score at the observed rate.

The MBTT score had an AuROC of 0.85 (95% CI: 0.78–0.91) which demonstrated good prediction (Figure 2). A Hosmer–Lemeshow goodness-of-fit test had a *p* value of 0.81 which demonstrated good calibration (Figure 3). Risk stratification was done according to its LH+ into low risk and high risk. In the high-risk group with a score of ≥4, LH+ was 6.72 (95% CI: 4.7–9.6, *p* < 0.001), and in the low-risk group of MBT in our study with a score of <4, LH+ was 0.4 (95% CI: 0.27–0.59). The risk stratification of prediction values of the scoring system is demonstrated in Table 3. Internal validation with bootstrap replications had an AuROC of 0.83 (95% CI: 0.75–0.91) which demonstrated good internal validation.

### 3.3. Comparison with Other Scoring Systems

**Figure 4:**
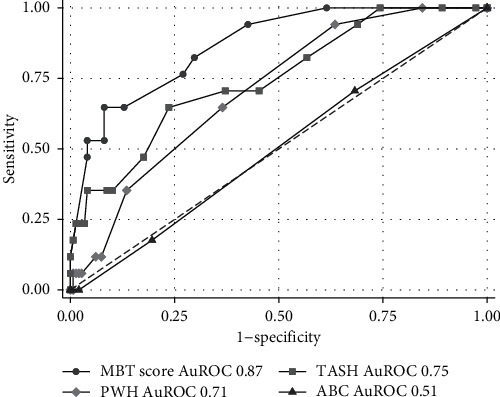
Comparison between the MBTT score and other scoring systems.

Other scoring systems, such as the ABC score, Prince of Wales Hospital (PWH) score, and Trauma-Associated Severe Hemorrhage (TASH) score, were validated with the same dataset as the MBTT score, and the results showed that the MBTT score had the highest AuROC. The second-best prediction was the PWH score with an AuROC of 0.71 (0.60–0.82), while the ABC score had the lowest AuROC of 0.51 (0.37–0.63). Comparisons between the MBTT score and the other scoring systems are shown in Figure 4.

## 4. Discussion

Our study included four significant parameters in the MBTT scoring system: age ≥60 years, BE ≤ –10 mEq/L, lactate >4 mmol/L, and HR ≥ 105/min. These parameters showed a good prediction performance with an AuROC of 0.845. All predictors in the model were reported to be associated with MBT in other studies (i.e., age [13, 14], BE [15], lactate level [16], and HR [13, 15, 17, 18].

The incidence of MBT in this study was 11.6%, while the incidences from previous studies were between 4.8% and 25% [6, 13, 17]. The incidence of MBT varied because those studies were conducted in different populations. Some studies reported the incidence in patients who met the trauma team activation criteria [6], while some studies reported patients who had high ISS scores [17]. A study that used the same population as our study was reported by Nunez et al. that reported an incidence of 12.5% which was similar with our study [6].

Compared with the scores that were previously published, our MBTT score had an AuROC of 0.845 which was close to the ABC score [6] that reported an AuROC of 0.842 and the TASH score [15] that had an AuROC of 0.892. The parameters in our study and in the ABC and TASH scores were also obtained rapidly after arrival. The AuROC in our study was lower than the modified Traumatic Bleeding Severity Score (TBSS) [13] which was 0.915 because the modified TBSS used more sophisticated parameters, such as type of pelvic fracture which may need a result from pelvic radiography, which take longer to obtain.

The ABC score and the TASH score had similar AuROC values in the original research. However, when those scores were validated in our study, the previously published scores had poor prediction. The explanation might be that the TASH score as well as the PWH score has hemoglobin as one of the predictors [15, 17]. In our study, hemoglobin was not included and was not significantly different between the MBT and non-MBT groups (12.6 mg/dL vs. 13.7 mg/dL), thus the decreased performance of the PWH and TASH scores. It must be noted that, in patients with acute blood loss, hemoglobin might not drop initially, especially in patients who do not receive a prehospital intravenous fluid infusion which is the case in Thailand.

In addition, the ABC score had a penetrating mechanism as one of the predictors [6]. In our sample, the predominant mechanism of injury was blunt force trauma. Penetrating mechanisms consist of GSW and stab wound. A stab wound causes less damage compared with a GSW. Our data revealed that 18% of patients who had GSW received MBT, while only 5% of patients who had stab wound received MBT. Therefore, when the ABC score included these two mechanisms of injury into one parameter, it decreased the power of discrimination.

The strength of this research lies in the fact that the data were collected from a trauma registry that were recorded prospectively. Therefore, the data were complete with only a few missing values. Since the parameters in this scoring system are easy to collect, this score is easy to apply in clinical practice. The limitation of the MBTT score is the availability of the lactate and BE measurements. Even though these two parameters are point-of-care tests, they are not available in all hospitals. Also, this study also needs an external validation in the future.

## 5. Conclusions and Clinical Applications

Our MBTT score consisted of four initial parameters that can be obtained immediately after arrival. With a MBTT score of <4, LH+ was 0.4 and the posttest probability was 0.04 which indicated that MBT would be rarely required. With a score of ≥4, LH+ was 6.72, in which case we would recommend preparing blood components.

## Figures and Tables

**Table 1:** Patient characteristics and univariable analysis of possible predictive parameters between the MBT and non-MBT groups.

	MBT (*N* = 102)	Non-MBT (*N* = 765)	OR	95% CI	*p* value	AuROC
Age, year, median (IQR)	33.5 (25–54)	33 (23–47)	1.01	0.99–1.02	0.10‡	0.55 (0.49–0.61)
Age ≥60 years, *n* (%)	21 (20.59)	85 (11.11)	2.07	1.22–3.52	0.006	0.55 (0.51–0.59)
Male, *n* (%)	77 (75.5)	634 (82.7)	1.57	0.96–2.56	0.08†	0.54 (0.49–0.58)
Mechanism of injury, *n* (%)			0.90	0.83–0.98	0.048	0.43 (0.38–0.48)
MCC	56 (54.9)	341 (44.6)				
MVC	11 (10.8)	88 (11.5)				
Bicycle	1 (0.98)	7 (0.9)				
AVP	6 (5.9)	29 (3.8)				
GSW	9 (8.8)	39 (5.1)				
SW	6 (5.8)	122 (15.9)				
Fall	12 (11.8)	97 (12.7)				
Assault	0 (0)	25 (3.3)				
Others	1 (1)	17 (2.2)				
SBP (mmHg), mean (SD)	119.8 (46.4)	131.5 (31.5)	0.99	0.98–0.99	0.0009§	0.4 (0.34–0.48)
SBP ≤90, *n* (%)	23 (22.6)	72 (9.4)	2.80	1.66–4.73	<0.001†	0.57 (0.52–0.61)
GCS score, median (IQR)	10 (4–15)	14 (8–15)	0.92	0.88–0.96	<0.001‡	0.39 (0.33–0.47)
GCS ≤8, *n* (%)	45 (44.1)	262 (34.3)	1.51	1.0–2.30	0.06†	0.55 (0.5–0.6)
HR (/min), mean (SD)	108 (33)	99 (26)	1.01	1.01–1.02	0.0012§	0.59 (0.53–0.66)
HR ≥ 105, *n* (%)	61 (59.8)	301 (38.8)	2.34	1.54–3.57	<0.001†	0.60 (0.55–0.66)
BE (mEq/L), median (IQR)	–10.1 (–13.1 to −5.9)	–4.7 (–7.4 to −2.1)	0.86	0.81–0.91	0.004‡	0.26 (0.18–0.35)
BE ≤ –10, *n* (%)	24 (52.2)	54 (10.7)	9.09	4.78–17.31	<0.001†	0.71 (0.63–0.78)
Lactate (mmol/L), mean (SD)	5.1 (3.7–6.8)	2.6 (1.7–4)	1.30	1.18–1.42	<0.001‡	0.76 (0.69–0.84)
Lactate >4, *n* (%)	38 (70.4)	132 (24)	7.54	4.07–13.95	<0.001†	0.73 (0.67–0.8)
Presence of free fluid from FAST, *n* (%)	37 (38.9)	129 (19.5)	2.63	1.67–4.14	<0.001†	0.6 (0.55–0.65)
Suspected pelvic fracture, *n* (%)	14 (13.7)	14 (1.8)	8.53	3.94–18.49	<0.001†	0.56 (0.53–0.59)
Suspected femur fracture, *n* (%)	16 (15.7)	30 (3.9)	4.56	2.38–8.70	<0.001†	0.56 (0.52–0.59)
Posterior nasal packing, *n* (%)	6 (5.9)	11 (1.4)	4.28	1.55–11.85	0.02†	0.52 (0.5–0.55)
PRBCs in 24 h (unit), median (IQR)	17 (13–25)	0 (0–2)	–	–	<0.001‡	–
FFP in 24 h (mL), median (IQR)	3539.5 (2197–5861)	0 (0–554)	–	–	<0.001‡	–
PC in 24 h (unit), median (IQR)	12 (6–23)	0 (0–0)	–	–	<0.001‡	–
ISS (IQR)	32 (22–38)	14 (8–25)	–	–	<0.001‡	–

MBT, massive blood transfusion; OR, odds ratio; CI, confidence interval; AuROC, area under the receiver operating characteristic; IQR, interquartile range; MCC, motorcycle crash; MVC, multivehicle crash; AVP, auto versus pedestrian; GSW, gunshot wound; SW, stab wound; SBP, systolic blood pressure; SD, standard deviation; GCS, Glasgow Coma Scale; HR, heart rate; BE, base excess; FAST, Focused Assessment Sonography in Trauma; PRBCs, packed red blood cells; FFP, fresh frozen plasma; PC, platelet concentrate; ISS, injury severity score. ^‡^Rank-sum test, ^†^exact probability test, ^§^*t*-test, and ^¶^chi-square test.

**Table 2:** Derivation of the scores from multivariable logistic regression analysis.

Parameters	OR	95% CI	*p* value	Coefficient	Score
Age ≥60 years	11.8	4.79–28.97	<0.001	2.33	3
BE ≤ –10 mEq/L	4.87	2.11–11.24	<0.001	1.70	2.5
Lactate >4 mmol/L	4.27	1.85–9.84	0.001	1.42	2
Heart rate ≥105/min	2.25	1.05–4.85	0.038	0.74	1

OR, odds ratio; CI, confidence interval; BE, base excess.

**Table 3:** Risk stratification of prediction values of the MBT scoring system.

Risk classification	Scores	Sensitivity (%)	Specificity (%)	Correctly classified (%)	LR+ (95% CI)	Posttest probability (95% CI)
Low	<4	36.4	9.5	11.7	0.4 (0.27–0.59)	0.04 (0.02–0.06)
High	≥4	63.6	90.5	88.3	6.72 (4.7–9.6)	0.61 (0.45–0.75)

LR+, positive likelihood ratio; CI, confidence interval.

## Data Availability

The data used to support the findings of this study are available from the corresponding author upon request.

## References

[1] Murray C. J. L., Vos T., Lozano R. (2012). Disability-adjusted life years (DALYs) for 291 diseases and injuries in 21 regions, 1990-2010: a systematic analysis for the Global Burden of Disease Study 2010. *Lancet*.

[2] Kauvar D. S., Wade C. E. (2005). The epidemiology and modern management of traumatic hemorrhage: US and international perspectives. *Critical Care*.

[3] Holcomb J. B., Tilley B. C., Baraniuk S. (2015). Transfusion of plasma, platelets, and red blood cells in a 1:1:1 vs a 1:1:2 ratio and mortality in patients with severe trauma: the PROPPR randomized clinical trial. *JAMA*.

[4] Cotton B. A., Dossett L. A., Haut E. R. (2010). Multicenter validation of a simplified score to predict massive transfusion in trauma. *The Journal of Trauma and Acute Care Surgery*.

[5] Poon K. M., Lui C. T., Tsui K. L. (2012). Comparison of the accuracy of local and international prediction models for massive transfusion in major trauma patients. *Hong Kong Journal of Emergency Medicine*.

[6] Nunez T. C., Voskresensky I. V., Dossett L. A., Shinall R., Dutton W. D., Cotton B. A. (2009). Early prediction of massive transfusion in trauma: simple as ABC (assessment of blood consumption)?. *The Journal of Trauma: Injury, Infection, and Critical Care*.

[7] Chaochankit W., Akaraborworn O., Sangthong B., Thongkhao K. (2018). Combination of blood lactate level with assessment of blood consumption (ABC) scoring system: a more accurate predictor of massive transfusion requirement. *Chinese Journal of Traumatology*.

[8] Wuthisuthimethawee P., Sookmee W., Damnoi S. (2019). Non-randomized comparative study on the efficacy of a trauma protocol in the emergency department. *Chinese Journal of Traumatology*.

[9] Patil V., Shetmahajan M. (2014). Massive transfusion and massive transfusion protocol. *Indian Journal of Anaesthesia*.

[10] ATLS Subcommittee (2018). *Advanced Trauma Life Support (ATLS®) Student Course Manult*.

[11] Moons K. G. M., Altman D. G., Reitsma J. B. (2015). Transparent Reporting of a multivariable prediction model for Individual Prognosis or Diagnosis (TRIPOD): explanation and elaboration. *Annals of Internal Medicine*.

[12] Chowdhury M. Z. I., Turin T. C. (2020). Variable selection strategies and its importance in clinical prediction modelling. *Family Medicine and Community Health*.

[13] Ogura T., Lefor A. K., Masuda M., Kushimoto S. (2016 Jun). Modified traumatic bleeding severity score: early determination of the need for massive transfusion. *The American Journal of Emergency Medicine*.

[14] Ogura T., Nakamura Y., Nakano M. (2014). Predicting the need for massive transfusion in trauma patients: the Traumatic Bleeding Severity Score. *The Journal of Trauma and Acute Care Surgery*.

[15] Yucel N., Lefering R., Maegele M. (2006). Trauma associated severe hemorrhage (TASH)-Score: probability of mass transfusion as surrogate for life threatening hemorrhage after multiple trauma. *Journal of Trauma-Injury Infection*.

[16] Brooke M., Yeung L., Miraflor E., Garcia A., Victorino G. P. (2016). Lactate predicts massive transfusion in hemodynamically normal patients. *Journal of Surgical Research*.

[17] Rainer T. H., Ho A. M.-H., Yeung J. H. H. (2011). Early risk stratification of patients with major trauma requiring massive blood transfusion. *Resuscitation*.

[18] Vandromme M. J., Griffin R. L., McGwin G., Weinberg J. A., Rue L. W., Kerby J. D. (2011). Prospective identification of patients at risk for massive transfusion: an imprecise endeavor. *The American Surgeon*.

